# DYNAMIC cohort study evaluating metabolic predictors of influenza vaccine immune response in older adults

**DOI:** 10.1038/s41541-022-00548-z

**Published:** 2022-11-01

**Authors:** Sapna P. Sadarangani, Barnaby E. Young, Weixiang Lian, Hwee Pin Phua, Mark I.-C. Chen, Ian Barr, Tsin Wen Yeo, Rinkoo Dalan, Angela Chow

**Affiliations:** 1 National Centre for Infectious Diseases, Singapore, Singapore; 2 Department of Infectious Diseases, Tan Tock Seng Hospital, Singapore, Singapore; 3 Lee Kong Chian School of Medicine, Nanyang Technological University, Singapore, Singapore; 4 Department of Preventive and Population Medicine, Office of Clinical Epidemiology, Analytics and Knowledge (OCEAN), Tan Tock Seng Hospital, Singapore, Singapore; 5 National Public Health Epidemiology Unit, Singapore, Singapore; 6 WHO Collaborating Centre for Reference and Research on Influenza, The Peter Doherty Institute for Infection and Immunity, Melbourne, VIC Australia; 7 Department of Endocrinology, Tan Tock Seng Hospital, Singapore Singapore,

**Keywords:** Translational research, Inactivated vaccines, Influenza virus

## Abstract

Immunosenescence (age-related immune dysfunction) and inflamm-aging contribute to suboptimal immune responses in older adults to standard-dose influenza vaccines, which may be exacerbated in those with metabolic co-morbidities. We sought to investigate metabolic factors/predictors of influenza vaccine immune response in an older adult (age ≥65 years) cohort in Singapore, where influenza typically circulates year-round. The primary outcome for the DYNAMIC prospective cohort study was haemagglutination-inhibition titer (HAI) response to each of the trivalent inactivated influenza vaccine strains at day 28 (D28) compared to baseline (D0), as assessed by seroconversion and D28/D0 log2 HAI fold rise. Baseline blood samples were tested for total Vitamin D (25-(OH) D) levels. We enrolled 234 participants in June–Dec 2017. Two hundred twenty completed all study visits. The median age was 71 [IQR 68–75] years, 67 (30.5%) had diabetes mellitus (DM), and the median BMI was 24.9 [IQR 22.2–27.8] kg/m^2^. Median baseline totals 25-(OH) D was 29 [IQR: 21–29] ng/ml. Age, DM, obesity, and baseline 25-(OH) D were not associated with HAI fold rise in multivariable analysis. More recent prior influenza vaccination and higher baseline HAI titers were associated with lower HAI fold rise for influenza A/HK/H3N2. Physical activity was associated with a higher HAI fold rise for influenza A/HK/H3N2 in a dose-response relationship (p-test for trend = 0.015). Older adults with well-controlled metabolic co-morbidities retain HAI response to the influenza vaccine, and physical activity had a beneficial effect on immune response, particularly for influenza A/HK/H3N2.

## Introduction

Prior to the COVID-19 pandemic and the associated non-pharmaceutical public health measures, influenza virus infection accounted for a significant burden of morbidity, hospitalization, and deaths annually, particularly among older adults^1,2^. Singapore, a tropical city-state with 5.7 million people, normally experiences year-round influenza circulation, with peaks coinciding with northern and southern hemisphere winters accounting for disproportionate deaths in persons older than 75 years^3^.

While there are ongoing efforts for an effective, durable, ‘universal’ influenza vaccine, the reduced effectiveness of standard-dose seasonal influenza protein subunit vaccines with haemagglutinin and neuraminidase antigens in older adults is well-recognized. Goodwin et al. estimated an adjusted odds-ratio of protective vaccine response in older versus young adults ranged from 0.24 to 0.59, defined by seroconversion and seroprotection as measured by haemagglutination-inhibition (HAI) titers^4^. This reduced effectiveness is partly due to age-related immune dysfunction, characterized by various changes in the innate and adaptive immune system, notably a reduced naïve T-cell pool, B-cell numbers, and inflamm-aging^5,6^.

Compounding these biological factors is the rising burden of metabolic co-morbidities in high- and low-middle-income countries (LMICs)^7,8^. Several studies in Asian populations have shown higher cardiometabolic risk and a higher body-fat proportion at lower BMI (body-mass index), thereby a World Health Organisation expert panel supported Asian countries implementing public health measures at lower BMI (BMI ≥ 23.0 kg/m^2^ for overweight/increased risk and BMI ≥ 27.5 kg/m^2^ for obesity/high risk) and incorporating waist circumference (central obesity) as an additional measure. The waist-circumference cutoffs used in Asian populations are also correspondingly lower (90 cm for men and 80 cm for women)^9,10^.

Both type 2 diabetes mellitus (DM) and obesity are associated with a higher risk of influenza-related hospitalization^11^. Importantly, these metabolic co-morbidities may contribute to inflamm-aging and the suboptimal response to vaccines^12^. The reported effects of DM on humoral (HAI) immune response to influenza vaccination are variable^13–16^. Among these studies, worsening glycemic control, increasing obesity (higher waist circumference), and lipids levels were associated with poorer HAI response^14,16–19^. However, there is a paucity of data on these measures from tropical and Asian countries despite high rates and rapidly rising prevalence of metabolic syndrome and obesity.

Vitamin D has complex immunomodulatory effects, specifically a robust action on the innate immune response, effects on cellular immunity with a bias towards a Th2 phenotype, and enhanced autoregulatory function of T-reg cells^20^. Although the Vitamin D receptor (VDR) is expressed widely in many human tissues and immune cells^20,21^, the exact threshold of systemic 25-hydroxyvitamin D (25-(OH) D) for its immune actions is unknown^22^. Animal studies have shown superior antigen-specific immune response for 1,25-(OH)_2_ D3-co-administered vaccines^23–25^. Prior human studies evaluating Vitamin D in the immune response to influenza vaccine had mixed results and were limited to selected populations^20,26^.

Our study aimed to evaluate the metabolic predictors and factors associated with influenza vaccine-specific adaptive immune response in older adults aged 65 and older living in the community in Singapore, as measured by HAI responses at day 28 post-trivalent inactivated influenza vaccine (IIV3) compared to baseline in a prospective cohort study. The factors of interest were baseline (day 0) 25-(OH) D levels, metabolic co-morbidities such as diabetes mellitus, obesity, and physical activity. We hypothesized that older persons with metabolic co-morbidities, obesity, or lower baseline 25-(OH) D levels would have a poorer immune response to the influenza vaccine.

## Results

### Study participant characteristics

**Fig. 1:**
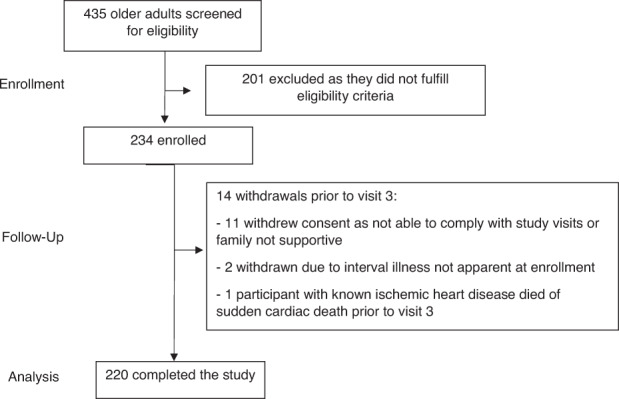
Study participant flow diagram for the DYNAMIC cohort study showing the enrollment, follow-up and analysis phases.

Four hundred and thirty-five participants were screened, and 234 were enrolled following written informed consent. Fourteen participants (6.0%) withdrew from the study before study completion (Fig. 1). Two hundred and twenty participants (94.0%) completed all study visits and composed the full analysis set. There were no significant baseline differences between the included and excluded participants. The median age for included 220 participants was 71 (IQR 68–75 and range 65–89) years, 64 (29.1%) were males, and 196 (89.1%) had at least one chronic condition. Specifically, 67 (30.5%) had diabetes mellitus, and 150 (68.2%) had hyperlipidemia. The median BMI was 24.9 [IQR 22.2–27.8] kg/m^2^ with a range of 15.2–49.9 kg/m^2^. The classifications of BMI in categories of normal, overweight, and obesity is based on cardiometabolic risk. Based on the WHO recommendation for the classification of obesity in Asian populations, 91 (41.4%) were overweight (BMI 23.0–27.4), and 62 (28.2%) were obese (BMI ≥ 27.5). The various ethnic groups in Singapore were represented in our enrolled population. Ninety-seven participants (44.1%) had received IIV3 in the 5 years before study enrollment.

### Geometric mean titers, seroprotection, and seroconversion

**Fig. 2:**
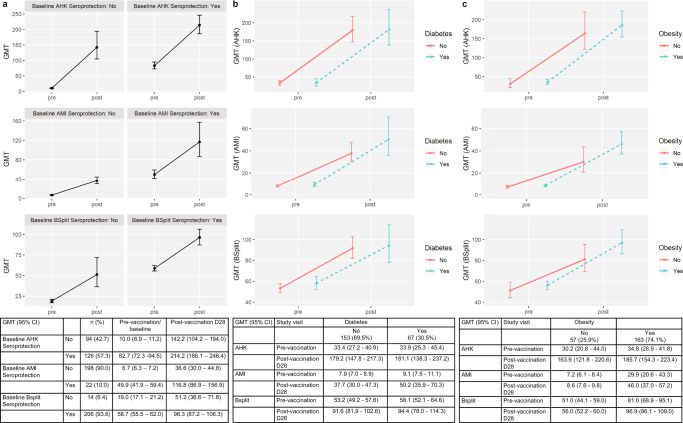
Geometric mean titers (GMT) pre-and post-influenza vaccine among full cohort for influenza A/HK/H3N2, A/MI/H1N1, and B-split, and among those with and without diabetes mellitus and obesity. Panels **a** shows line graphs depicting GMT and 95% confidence intervals pre- and post-influenza vaccination for A/HK/H3N2, A/MI/H1N1 and B-split for the full cohort, subcategorized by their baseline seroprotection status (HAI of at least 1:40) for each respective strain. Panels **b** shows GMT and 95% confidence intervals pre- and post-influenza vaccination for the three strains amongst those with diabetes mellitus (*n* = 67) compared to those without. Panels **c** show GMT and 95% confidence intervals pre-and post-influenza vaccination for the three strains amongst those with obesity (*n* = 163, applying the same definition as in Tables 1 and 2) compared to those without. The embedded table displays the GMT and 95% CI data as shown in the corresponding panel line graphs.

The geometric mean titers for each vaccine strain at baseline and post-vaccination for the full cohort and comparing those with and without diabetes mellitus and obesity are shown in Fig. 2. 214 (97.3%) participants were seroprotected to at least one strain at baseline compared to 219 (99.5%) at D28 (titer of ≥1:40); specifically 126 (57.3%) at baseline for A/HK/H3N2 and 205 (93.2%) at D28, 22 (10.0%) at baseline for A/MI/H1N1 and 133 (60.5%) at D28, 206 (93.6%) at baseline for influenza B and 218 (99.1%) at D28. High baseline titers and seroprotection could result from prior influenza vaccination or natural infection. The lower baseline seroprotection for A/MI/H1N1 in comparison to influenza A/H3N2 and influenza B is expected as the circulating strain in the immediate previous season had been A/H1N1/California/7/2009, which had been included in the previous season’s vaccines (Supplementary Information). Due to high baseline seroprotection rates for two of the three strains, the D28 seroprotection rate is less meaningful as an outcome, hence we focused on seroconversion at D28 and D28/baseline log2 HAI fold rise for our analyses.

One hundred sixty-nine (76.8%) seroconverted to at least one of the three strains contained in the vaccine, with 124 (56.4%) seroconverting to influenza A/HK/H3N2, 110 (50.0%) to A/MI/H1N1, and 34 (15.5%) to influenza B. Table 1 shows the clinical and demographic variables amongst participants who seroconverted to each strain and those who did not. Among those who seroconverted to at least one strain, a lower proportion had received a more recent influenza vaccine (SH 2016 or NH 2016–2017 vaccine) as compared to those who did not seroconvert (26.0% vs 60.8%). Those who seroconverted to at least one strain had a lower pre-vaccine titer as compared to those who did not. This was most pronounced for influenza A/HK/H3N2, where the 2017 study vaccine had similar antigen composition compared to the immediate previous season’s vaccine (Supplementary Information). Women and younger participants were more likely to seroconvert to at least one strain.

**Table 1:** Socio-demographic, medical and influenza vaccination history, physical activity, and baseline titers, by seroconversion status to each of the vaccine strains.

	Overall	A/HK/H3N2 seroconversion			A/MI/H1N1 seroconversion			B-Split seroconversion			Any seroconversion		
		Yes	No	*p*	Yes	No	*p*	Yes	No	*p*	Yes	No	*p*
*n*	220	124 (56.4)	96 (43.6)		110 (50.0)	110 (50.0)		34 (15.5)	186 (84.5)		169 (76.8)	51 (23.2)	
Age, years	71.0 [68.0, 75.0]	70.0 [68.0, 74.3]	72.0 [68.0, 77.0]	0.073	71.0 [68.0, 74.8]	71.50 [68.3, 76.0]	0.173	71.50 [67.3, 76.0]	71.0 [68.0, 75.0]	0.770	71.0 [68.0, 75.0]	74.0 [69.0, 77.0]	0.028
Gender (male)	64 (29.1)	30 (24.2)	34 (35.4)	0.069	27 (24.5)	37 (33.6)	0.138	10 (29.4)	54 (29.0)	0.964	42 (24.9)	22 (43.1)	0.012
Ethnicity				0.244			0.018						0.348
Chinese	142 (64.5)	77 (62.1)	65 (67.7)		61 (55.5)	81 (73.6)		22 (64.7)	120 (64.5)	0.179	105 (62.1)	37 (72.5)	
Malay	45 (20.5)	24 (19.4)	21 (21.9)		29 (26.4)	16 (14.5)		4 (11.8)	41 (22.0)		36 (21.3)	9 (17.6)	
Indian/others^a^	33 (15.0)	23 (18.5)	10 (10.4)		20 (18.2)	13 (11.8)		8 (23.5)	25 (13.4)		28 (16.6)	5 (9.8)	
BMI (kg/m^2^)				0.117			0.388			0.166			0.792
<18.5 (underweight)	9 (4.1)	6 (6.2)	3 (2.4)		3 (2.7)	6 (5.5)		8 (4.3)	1 (2.9)		2 (3.9)	7 (4.1)	
18.5–22.9 (normal)	58 (26.4)	27 (28.1)	31 (25.0)		32 (29.1)	26 (23.6)		48 (25.8)	10 (29.4)		13 (25.5)	45 (26.6)	
23.0–27.4 (overweight)	91 (41.4)	32 (33.3)	59 (47.6)		41 (37.3)	50 (45.5)		82 (44.1)	9 (26.5)		19 (37.3)	72 (42.6)	
≥27.5 (obese)	62 (28.2)	31 (32.3)	31 (25.0)		34 (30.9)	28 (25.5)		48 (25.8)	14 (41.2)		17 (33.3)	45 (26.6)	
Waist/ hip/ BMI^b^	163 (74.1)	92 (74.2)	71 (74.0)	0.968	83 (75.5)	80 (72.7)	0.644	26 (76.5)	137 (73.7)	0.731	123 (72.8)	40 (78.4)	0.42
Diabetes	67 (30.5)	39 (31.5)	28 (29.2)	0.715	35 (31.8)	32 (29.1)	0.66	9 (26.5)	58 (31.2)	0.583	51 (30.2)	16 (31.4)	0.871
Hypertension	144 (65.5)	78 (62.9)	66 (68.8)	0.366	69 (62.7)	75 (68.2)	0.395	22 (64.7)	122 (65.6)	0.92	108 (63.9)	36 (70.6)	0.379
Hyperlipidemia	150 (68.2)	82 (66.1)	68 (70.8)	0.458	73 (66.4)	77 (70.0)	0.563	23 (67.6)	127 (68.3)	0.942	112 (66.3)	38 (74.5)	0.268
Chronic pulmonary disease	10 (4.5)	1 (0.8)	9 (9.4)	0.003	4 (3.6)	6 (5.5)	0.517	2 (5.9)	8 (4.3)	0.655	5 (3.0)	5 (9.8)	0.054
At least one chronic condition	196 (89.1)	111 (89.5)	85 (88.5)	0.818	98 (89.1)	98 (89.1)	1.000	32 (94.1)	164 (88.2)	0.386	150 (88.8)	46 (90.2)	0.773
Physical activity				0.477			0.141			0.442			0.860
Rare	13 (5.9)	5 (4.0)	8 (8.3)		8 (7.3)	5 (4.5)		2 (5.9)	11 (5.9)		11 (6.5)	2 (3.9)	
Light	109 (49.5)	64 (51.6)	45 (46.9)		60 (54.5)	49 (44.5)		18 (52.9)	91 (48.9)		84 (49.7)	25 (49.0)	
Intermediate	22 (10.0)	12 (9.7)	10 (10.4)		8 (7.3)	14 (12.7)		5 (14.7)	17 (9.1)		15 (8.9)	7 (13.7)	
Moderate	62 (28.2)	33 (26.6)	29 (30.2)		25 (22.7)	37 (33.6)		9 (26.5)	53 (28.5)		48 (28.4)	14 (27.5)	
Vigorous	14 (6.4)	10 (8.1)	4 (4.2)		9 (8.2)	5 (4.5)		0 (0.0)	14 (7.5)		11 (6.5)	3 (5.9)	
Baseline 25-(OH)-D (ng/mL)	26.0 [21.0, 29.0]	26 [21, 29]	26.0 [21.0, 29.3]	0.852	25.5 [20.3, 29.0]	26.0 [22.0, 30.0]	0.140	26.0 [22.3, 28.8]	26.0 [21.0, 29.0]	0.922	26.0 [21.0, 29.0]	26.0 [21.5, 29.5]	0.924
History of influenza vaccination				<0.001			0.483			0.099			<0.001
None in past 5 years	123 (55.9)	89 (71.8)	34 (35.4)		63 (57.3)	60 (54.5)		25 (73.5)	98 (52.7)		108 (63.9)	15 (29.4)	
SH 2014-NH 2014/15 SH 2015-NH 2015/16^c^	22 (10.0)	15 (12.1)	7 (7.3)		13 (11.8)	9 (8.2)		2 (5.9)	20 (10.8)		17 (10.1)	5 (9.8)	
SH 2016-NH 2016/17^c^	75 (34.1)	20 (16.1)	55 (57.3)		34 (30.9)	41 (37.3)		7 (20.6)	68 (36.6)		44 (26.0)	31 (60.8)	
Baseline (pre-vaccine) A/HK/H3N2 titer	40 [10, 80]	20 [10, 40]	80 [40, 160]	<0.001	40 [10, 80]	40 [10, 80]	0.656	40 [10, 80]	40 [10, 80]	0.801	40 [10, 80]	80 [40, 160]	<0.001
Baseline (pre-vaccine) A/MI/H1N1 titer	5 [5, 10]	5 [5, 10]	7.50 [5, 20]	<0.001	5 [5, 10]	5 [5, 10]	0.145	5 [5, 10]	5 [5, 10]	0.35	5 [5, 10]	5 [5, 20]	0.080
Baseline (pre-vaccine) B-split titer	40 [40, 80]	40 [40, 80]	80 [40, 80]	0.020	60 [40, 80]	40 [40, 80]	0.523	40 [40, 80]	40 [40, 80]	0.323	40 [40, 80]	80 [40, 80]	0.436

Continuous variables are reported as Median [IQR] while categorical variables are reported as Counts (%). Seroconversion is defined by at least a four-fold rise and antibody titer of at least 40.

^a^Others group comprised one Eurasian participant who was ethnically closest to the Indian subgroup.

^b^Waist/Hip/BMI—

Any one of the conditions below fulfilled: (Yes, No).

• Waist circumference >90 cm for men.

• Waist circumference >80 cm for women.

• Waist: hip ratio >0.90 for men.

• Waist: hip ratio >0.85 for women.

• BMI > 27.5 kg/m2 for both men and women.

^c^SH (southern hemisphere) vaccine and NH (northern hemisphere) vaccine.

^d^Physical activity assessed by Rapid Assessment of Physical Activity—RAPA questionnaire (Aerobic):

• Rare (or sedentary)—rare or never do physical activity.

• Light—light or moderate physical activity, but not every week, or do some light activity every week.

• Intermediate—moderate physical activity every week <30 min/day, 5 days/week, OR vigorous activities every week but less than 20 min a day, 3 days/week.

• Moderate—moderate physical activity ≥30 min/day, ≥5 days a week.

• Vigorous—vigorous physical activity ≥20 min/day ≥3 days a week.

### Multivariable analyses for HAI fold rise

In the multivariable analyses, increasing age, gender, and ethnicity were not significantly associated with HAI fold rise at D28. Similarly, anthropometric markers of obesity, co-morbidities, and baseline 25-(OH) D were also not found to be significantly associated with HAI fold rise (Table 2). We used a composite variable for obesity in our regression model, combining waist circumference, waist-hip circumference ratio, and obese BMI to avoid multicollinearity while enhancing sensitivity. While the threshold for 25-(OH) D’s immune actions is not defined, we observed a reasonably wide range of levels in our participants (10–42 ng/ml). Physical activity was significantly associated with a notable increase in HAI fold rise for influenza A/HK/H3N2 in a dose-response relationship generally, where there is an overall tendency for participants reporting a higher level of physical activity to have a higher increase in HAI fold rise for influenza A/HK/H3N2 (p for trend = 0.015). Participants reporting light physical activity levels had 2.04 times [95% CI 1.06–3.91] higher A/HK/H3N2 fold rise as compared to those who reported being rarely involved in physical activity. The lack of dose-response relationship seen among patients with vigorous physical activity could be due to the small sample size in this group (*n* = 14). Participants with more recent prior influenza vaccine had significantly lower HAI fold rise for influenza A/HK/H3N2 as compared to those with no prior influenza vaccination in past 5 years (*p* < 0.001).

**Table 2:** Multivariable regression analyses of factors associated with A/HK/H3N2, A/MI/H1N1, and B HAI fold rise at D28 compared to D0.

Variables	A/HK/H3N2 fold rise (95% CI)	*p*	A/MI/H1N1 fold rise (95% CI)	*p*	B-split fold rise (95% CI)	*p*
Age	0.99 (0.96–1.02)	0.571	0.96 (0.92–1.00)	0.036	1.00 (0.98–1.02)	0.941
Gender						
Female	Ref	—	Ref	—	Ref	—
Male	0.76 (0.55–1.07)	0.120	0.72 (0.48–1.08)	0.116	1.01 (0.83–1.23)	0.921
Ethnicity						
Chinese	Ref	—	Ref	—	Ref	—
Malay	1.14 (0.74–1.75)	0.561	1.36 (0.81–2.28)	0.240	0.96 (0.74–1.24)	0.746
Indian/others^a^	1.40 (0.89–2.20)	0.145	1.66 (0.97–2.84)	0.068	1.23 (0.94–1.61)	0.134
Waist/ hip/ BMI^b^	1.11 (0.77–1.60)	0.563	1.32 (0.86–2.04)	0.206	1.12 (0.90–1.39)	0.302
Diabetes	1.10 (0.78–1.56)	0.594	1.12 (0.74–1.69)	0.606	1.00 (0.81–1.23)	0.988
Hypertension	0.85 (0.59–1.22)	0.389	0.91 (0.59–1.41)	0.682	0.93 (0.75–1.16)	0.539
Hyperlipidemia	0.89 (0.62–1.28)	0.532	0.69 (0.45–1.07)	0.101	1.01 (0.81–1.25)	0.959
Chronic pulmonary disease	0.58 (0.28–1.20)	0.143	0.91 (0.38–2.16)	0.829	0.97 (0.63–1.49)	0.895
Physical activity		0.015^d^		0.056^d^		0.082^d^
Rare	Ref	—	Ref	—	Ref	—
Light	2.04 (1.06–3.91)	0.034	0.97 (0.44–2.11)	0.932	0.97 (0.66–1.43)	0.883
Intermediate	2.52 (1.14–5.57)	0.023	0.67 (0.26–1.72)	0.401	1.14 (0.71–1.82)	0.596
Moderate	2.78 (1.41–5.51)	0.004	0.60 (0.26–1.35)	0.219	0.92 (0.61–1.38)	0.690
Vigorous	2.53 (1.07–5.99)	0.036	0.81 (0.29–2.28)	0.694	0.60 (0.36–1.00)	0.052
Baseline 25-(OH)-D ng/mL	1.00 (0.98–1.03)	0.753	1.00 (0.97–1.03)	0.843	1.00 (0.99–1.02)	0.725
History of Influenza vaccination						
None	Ref	—	Ref	—	Ref	—
SH 2014- NH 2014/15/ SH 2015-NH 2015/16^c^	0.69 (0.41–1.14)	0.148	1.71 (0.93–3.13)	0.085	0.87 (0.65–1.18)	0.378
SH 2016- NH 2016/17^c^	0.45 (0.31–0.66)	<0.001	1.29 (0.82–2.03)	0.269	0.78 (0.62–0.97)	0.029
Baseline A/HK/H3N2 titer (Log2 Transformed)	0.66 (0.60–0.73)	<0.001	1.03 (0.92–1.15)	0.591	1.05 (1.00–1.11)	0.063
Baseline A/MI/H1N1 titer (Log2 Transformed)	0.93 (0.80–1.07)	0.316	0.74 (0.62–0.87)	0.001	1.01 (0.92–1.10)	0.870
Baseline B-Split titer (Log2 Transformed)	1.08 (0.86–1.35)	0.497	1.10 (0.84–1.44)	0.502	0.81 (0.71–0.92)	0.002

All *p-*values obtained by testing if the coefficient for a particular level of variable is significantly different compared to the reference level chosen for that variable after adjustment for all other variables in the regression model.

^a^Others group comprised one Eurasian participant who was ethnically closest to the Indian subgroup.

^b^Waist/Hip/BMI—

Any one of the conditions below fulfilled: (Yes, No).

• Waist circumference >90 cm for men.

• Waist circumference >80 cm for women.

• Waist: hip ratio >0.90 for men.

• Waist: hip ratio >0.85 for women.

• BMI > 27.5 kg/m2 for both men and women.

^c^SH (southern hemisphere) vaccine and NH (northern hemisphere) vaccine.

^d^p-test for trend for physical activity.

In the models which included interaction terms between prior influenza vaccine type received and baseline HAI titer for fold rise in A/HK/H3N2, A/MI/H1N1, and B-Split, there is no statistically significant interaction found on the respective fold rise among prior influenza vaccination groups with different baseline HAI titers (Supplementary table 1).

## Discussion

Our findings in this cohort of 220 older adults with well-controlled chronic conditions living in the community showed that 169 (76.8%) seroconverted to at least one of the influenza vaccine strains contained in the vaccine. Age, diabetes mellitus, baseline 25-(OH) D, markers of obesity, were not significantly associated with HAI response based on seroconversion and HAI fold rise. Physical activity was associated with a higher A/HK/HAI fold rise in a general dose-response relationship. Our finding of prior seasonal influenza vaccination and high pre-vaccination titer associated with lower seroconversion/HAI fold rise (particularly if the prior season’s vaccine contained a congruent strain with the current season’s vaccine) is consistent with findings from other studies examining cohorts with prior and repeated influenza vaccination, particularly for A/H3N2^27–29^. Several authors have postulated this may be linked to the ‘antigenic distance’ hypothesis^27^. A more recent influenza vaccination (while accounting for waning) may also be associated with a higher baseline titer (57.3% of our cohort were seroprotected to A/H3N2 at baseline), although prior natural infection may also account for higher baseline titers. The immune response to the current vaccine might also differ if the prior vaccine received had congruent strains with the current vaccine. We, therefore, investigated this in the interaction terms between the prior influenza vaccine received and baseline titer but did not find any significant modifying effects on HAI response. As a comparison, Snape et al enrolled 75 COPD and 72 age-matched healthy older adults (range 50–90 years) in Brisbane and Melbourne, Australia, and assessed influenza vaccine immunogenicity via HAI seroconversion at Day 28 as the primary end-point^29^. Importantly, approximately 90% of this cohort had received recent influenza vaccination in the preceding two years. The authors found that the year of vaccine, vaccine strain, and study site were independently associated with seroconversion at Day 28 in multiple logistic regression. Age and BMI (median BMI 26.7 kg/m^2^, range 18.2–52.9) were not associated with seroconversion, generally similar to our study findings, and notably, the age range of participants was wider while the BMI range was comparable. The authors postulated site environmental variables and vitamin D status may have contributed, given the different latitudes/climates, but this needs to be studied further. In further analyses, the authors concluded the variation observed from the year of vaccination was attributable to the vaccine strain (higher seroconversion with novel strains) and higher baseline antibody levels negatively correlated with the amplitude of fold increase in post-vaccination antibody, similar to our findings.

Importantly, while studies have shown some impact of diabetes mellitus and obesity on influenza vaccine immune response, our study and others have shown that well-controlled co-morbidities may not have any significant effect on HAI response^13,14,29,30^. However, HAI titers have limitations concerning translation to protection from infection in older adults. While pre- and post-vaccination HAI titers are commonly used and accepted in immunogenicity studies, HAI titers do not fully explain protection from infection. Furthermore, HAI 1:40 represents 50% protection from infection based on studies performed in younger adults^31^. While there is no accepted correlate of protection, other wider immune responses, such as antibody-dependent cellular cytotoxicity and cell-mediated immunity, are important considerations.

Our finding of the beneficial association of physical activity on HAI response has also been shown by other investigators^32–34^. Importantly, physical activity retains benefits even after adjusting for BMI and co-morbidities, and we observed relative benefit even for modest physical activity. Wong et al. showed improved B cell and plasmablast expansion after vaccination in active compared to sedentary women^33^. The type of physical activity, and consistency over time likely influence the overall physiological benefits, and the mechanisms are not yet fully elucidated. The lack of regular physical activity and obesogenic environments is a great concern and anticipated major risk factors for global mortality^35^. Promoting physical activity is expected to have wider benefits.

Our study did not find any association of vitamin D with HAI response. Multivariable regression of HAI response has shown that there is no correlation in HAI fold-rise with unit increases in baseline 25-(OH) D level in any vaccine strain while controlling for other covariates. In vitro studies suggest vitamin D may modulate B-cell differentiation but does not stimulate plasma cells or memory B cells, and the lack of association of 25-(OH) D on humoral responses to influenza vaccine has been observed in other studies^18,24^. Nevertheless, the role of Vitamin D in vaccine response may still be relevant through other pathways, such as cell-mediated immunity^18^. In addition, a recent concept of the “personal vitamin D response index” related to molecular and epigenetic variations in the vitamin-D-signaling pathway may explain variable vitamin-D-responsiveness or ‘sufficiency thresholds’ for individuals and population groups^36^. This may potentially explain the conflicting results of vitamin D observational and supplementation studies. The “vitamin D-response index” could be studied in greater depth in conjunction with systemic Vitamin D levels or targeted supplementation once this is better defined.

Our study has notable strengths. We enrolled a community older adult cohort with well-represented Asian ethnic groups from Singapore with a representative proportion of participants with metabolic conditions such as diabetes mellitus, hyperlipidemia, and elevated BMI comparable with national rates^37,38^. Due to the rising prevalence of metabolic syndrome and obesity in Asian countries (both high-income and LMIC), our study contributes to the urgent need for well-designed studies in Asian populations to better understand the impact of metabolic co-morbidities on the immune response to vaccination (and infection) that will inform public health action. Vitamin D deficiency (based on bone health) is not uncommon in tropical countries. While there is no accepted minimum systemic Vitamin D level for its non-skeletal and immunomodulatory actions, it is important to study this in local populations. Our high study retention and completion help to minimize bias from loss-to-follow-up. HAI assays were performed at the WHO collaborating laboratory with standard protocols.

There are a few limitations that we need to acknowledge. Firstly, HAI response alone does not provide the full spectrum of the immune response. We are investigating cell-mediated immunity assays separately. Secondly, while other studies have found some impact of obesity and high BMI on influenza vaccine immune response, we were not able to replicate these findings. Whether this is due to study and population heterogeneity, or other population-level biological differences is unclear. We used more than one measure of obesity, i.e., BMI, waist, and hip circumference. Our findings may not apply to populations with a higher proportion of overweight or obese older adults. Further study with adipokines, examining for leptin or insulin resistance, and studies in populations with greater prevalence of obesity/severe obesity may be informative. Thirdly, we may be underpowered for certain analyses, for example, the analyses with interaction terms. Fourthly, while our study enrolled a multi-ethnic older adult Asian cohort, our findings would not be generalizable to a very frail population or to those with poorly controlled metabolic syndrome. Lastly, while we have interesting findings related to physical activity and HAI response, the data on physical activity was obtained via interviewer-administered questionnaires, although the RAPA questionnaire used has been validated in other settings in older adults^39^. We did not have directly observed data by active reporting or pedometers, and our data may be subject to recall and social desirability bias. However, the observed effects are likely to be real albeit attenuated, as the measurement error is likely to be non-differential and biased toward the null. Conversely, while intervention studies are viewed as a superior study design, there are pragmatic disadvantages to extrapolating prescribed scenarios in trial settings regarding physical activity habits to the population scale. Nonetheless, the congruence of our findings with other studies and the observed dose-response relationship provides more credibility.

In conclusion, our study found that older adults with well-controlled metabolic co-morbidities do retain HAI response to influenza vaccine, and physical activity had a beneficial effect on HAI fold rise, particularly for influenza A/H3N2. We recommend the encouragement of influenza vaccine uptake among older adults. Ideally, a novel influenza vaccine with more durable protection is desirable, either using mRNA technology or other platforms targeting more conserved regions of the influenza virus. Meanwhile, a personalized influenza vaccination approach has been suggested with the assessment of pre-vaccination antibody levels^29^, but the latter would be difficult to implement at the population scale without an assay widely available in clinical laboratories and a well-established correlate of protection. Physical activity is a potential modifiable beneficial low-cost lifestyle intervention for vaccine HAI response, along with other wider health benefits. Finally, we recommend older adults with metabolic conditions be specifically studied, including evaluating dynamic host factors that could optimize influenza vaccine effectiveness in this population.

## Methods

### Study design

**Fig. 3:**
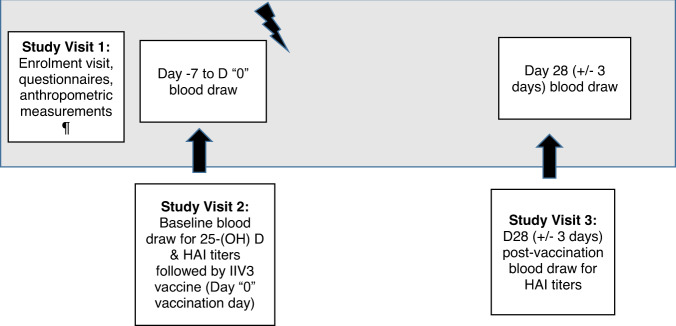
Outline of study visits, the DYNAMIC cohort study. ¶ anthropometric measurements comprised weight (kg), height (m), waist, and hip circumferences (cm). BMI (kg/m^2^), and waist: hip circumference ratios were calculated.

This prospective cohort study comprised three study visits shown in Fig. 3. Each enrolled participant had up to 23 milliliters (ml) of venous blood collected at two time-points, baseline and at D28 (±3 days) following intramuscular influenza vaccination. During the first study visit, participants completed an interviewer-administered questionnaire by trained interviewers with data collected on demographic information, medical history, vaccination history, and health and physical activity habits (Rapid Assessment of Physical Activity—RAPA questionnaire)^40^.

### Ethical approval

The study was approved by the National Healthcare Group Institutional Review Board (DSRB 2016/00248). At the time of the study’s enrollment, all human subject studies with an intervention (such as the influenza vaccine for this study) were regulated by Singapore’s Health Sciences Authority (this study’s Clinical Trial Notification CTN1700053 and ClinicalTrials.gov NCT03399357).

### Participants and recruitment

Adults aged 65 and older residing in the community in Singapore were prospectively enrolled after written informed consent at community sites and our institution’s research clinic from June to December 2017. In-person study visits concluded by February 2018. The community sites comprised a few sites that were part of the National Healthcare Group’s mid-year influenza vaccination program outreach for older adults, and other sites engaged in community initiatives for older adults (Supplementary Information).

Eligible older adults included those with well-controlled chronic conditions if they had not been hospitalized in the preceding 12 months. They should also have received their most recent annual seasonal influenza vaccine no more recently than 10 months prior. Immunocompromised adults were excluded. The detailed inclusion and exclusion criteria are shown in Box 1.

Box 1 Inclusion and exclusion Criteria for the DYNAMIC cohort study **Inclusion criteria**

- Age ≥65 years on the day of inclusion (not older than 100 years).
- Able to give written informed consent.
- Has NO immunosuppressive conditions and determined by medical evaluation, and clinical judgment to be generally healthy or have only stable medical conditions (no hospitalizations for worsening of disease in past 12 weeks prior to vaccination).
- Note: Change in the dose of medication or therapy within the treatment category is allowed. Change to new therapy only allowed if not due to worsening disease.
- Is eligible for seasonal influenza vaccine.
- Able to attend and willing to comply with all scheduled visits and comply with all study procedures.

**Exclusion criteria**

- A change to a new therapy category caused by worsening disease, or worsening chronic disease for any reason.
- Symptoms suggestive of influenza, influenza-like illness, or respiratory illness.
- Vaccination with any licensed or experimental influenza vaccine within the past 10 months.
- Intent to receive any other investigational vaccine or agent during the course of the study.
- Intent to receive other licensed vaccines during the course of the study (does not apply for a pandemic or post-exposure prophylaxis scenario).
- Allergic to egg proteins (egg or egg products) and chicken proteins (serious allergy).
- History of a life-threatening reaction to the vaccine used in the study, vaccine components (formaldehyde, cetylmethylammonium bromide (CTAB), polysorbate 80, gentamicin, or excipients such as potassium chloride) or to a vaccine containing any of the same substances.
- Known or suspected immunodeficiency or receiving treatment with immunosuppressive therapy including cytotoxic agents (in past 6 months) e.g., transplant recipients on active immunosuppression, patients with cancer, HIV, or autoimmune disease.
- Long-term systemic corticosteroid therapy (prednisolone ≥ 7.5 mg/day or equivalent for more than 2 consecutive weeks within the past 3 months).
- Note: If systemic corticosteroids have been administered short term for the treatment of an acute illness, subjects will be excluded from the study until corticosteroid therapy has been discontinued for at least 30 days.
- History of Guillain-Barré syndrome (GBS).
- Serious chronic illness that, in the opinion of the investigator, is at a severity or stage which might interfere with study conduct or completion.^a^
- Bariatric surgery, GI malabsorption disorders.
- Recurrent Falls (≥2 falls in the past 12 months).
- Osteoporosis with or without pathological fractures.
- Current or recently completed high-dose vitamin D supplementation within the past 3 months (defined as daily cholecalciferol dose of 2000 IU or higher, weekly 50,000 IU or intramuscular calcitriol).
- Receipt of any blood products, including immunoglobulin, within 6 months of study enrollment.
- Donated blood within the last 58 days.
- Current anticoagulant therapy or a history of bleeding diathesis (including thrombocytopenia with platelet count <50,000) that would contraindicate intramuscular (IM) injection (Note: antiplatelet drugs such as aspirin and clopidogrel are permitted).
- Moderate or severe acute illness/infection (according to investigator judgment) on the day of vaccination, or febrile illness (temperature ≥ 37.5 °C). A prospective subject should not be included in the study until the condition has resolved or the febrile event has subsided.
- Any medical condition or situation that would, in the opinion of the investigator, interfere with the evaluation of the study objectives.

^a^Serious chronic medical condition including: metastatic malignancy, a severe chronic obstructive pulmonary disease requiring supplemental oxygen, CKD stage 3 and above, end-stage renal disease with or without dialysis, clinically unstable cardiac disease, or any other disorder that, in the investigator’s opinion, should preclude the subject from participating in the study

### Vaccine

All participants received the southern hemisphere 2017 IIV3 Influvac® containing an A/Hong Kong/4801/2014-H3N2-like virus, A/Michigan/45/2015-H1N1, and B/Brisbane/60/2008^41^. The northern hemisphere IIV3 2017–18 composition was identical to the composition of the 2017 southern hemisphere vaccine^42^. Trivalent inactivated seasonal influenza vaccines were the standard clinically available vaccines in Singapore at the time of this study’s enrollment. The vaccine was maintained in a cold chain between +2 and +8 °C as recommended by the manufacturer before administration to participants via intramuscular injection in the deltoid muscle by a trained research nurse. Temperature monitoring was performed for vaccines administered at all study sites.

### Laboratory methods

Blood samples were stored at +2 to +8 °C following collection and delivered to a central laboratory at Singapore Immunology Network (SIgN) for processing. Peripheral blood mononuclear cells (PBMCs) and serum were stored at −80 °C and only thawed at the time of assays. Assays on PBMCs assessing markers of cell-mediated immunity will be reported separately.

### 25-hydroxyvitamin D (25-(OH) D) assay

25-Hydroxyvitamin D2 and 25-hydroxyvitamin D3 were measured on baseline sera for participants who completed all study visits at the Mayo Clinic Immunochemical Core Laboratory by liquid chromatography-tandem mass spectrometry (LC-MS/MS) (ThermoFisher Scientific, Franklin, Massachusetts 02038 and Applied Biosystems-MDS Sciex, Foster City, CA 94404). Intra-assay C.V.’s for 25-(OH) D2 were 4.4%, 3.3%, and 4.2% at 14, 41, and 124 ng/mL, respectively. Interassay C.V.’s were 6.1%, 6.2%, and 4.7% at 15, 43, and 128 ng/mL, respectively. Intra-assay C.V.’s for 25-(OH) D3 were 3.8%, 2.4%, and 4.7% at 25, 54, and 140 ng/mL, respectively. Interassay C.V.’s were 6.4%, 6.8%, and 5.0% at 24, 52, and 140 ng/mL respectively. As 25-(OH) D2 were universally low, the total 25-(OH) D was used for analyses.

### Haemagglutination-inhibition (HAI) assays

HAI assays were performed on the baseline and D28 sera at the WHO Influenza Collaborating Centre for Reference and Research on Influenza in Australia, using standard protocols with egg-grown influenza viruses contained in trivalent influenza inactivated vaccine the subjects had received, namely A/Hongkong/4801/2014-H3N2 like virus, A/Singapore/GP1908/2015-H1N1 (antigenically identical to the A/Michigan/45/2015-H1N1 contained in the vaccine) and B/Brisbane/60/2008^43^. The data used for the analyses for B/Brisbane/60/2008 were from assays performed with the ether-split virus. Turkey red blood cells were used for all assays. Additional dilutions were performed as needed for high HAI titers.

### Statistical analysis

We estimated the sample size based on the study objective for 25-(OH) D status on HAI response. In our null hypothesis, the proportion of vitamin-D-deficient and non-deficient subjects with HAI seroconversion would be the same. We obtained a conservative estimate of 30% to be vitamin D deficient for our study population based on existing literature, including studies investigating Vitamin D status/deficiency in Singapore^20,26,44–46^, and assumed 20% of vitamin-D-deficient patients and 40% of non-deficient patients will seroconvert to any of the influenza vaccine strains. To detect this difference in proportion with a Pearson’s Chi-square test, we set the level of significance at 5% and a power of 0.8 to derive a total sample size of 237 required for the study (Supplementary Table 2).

The HAI titers appear in results as A/HK/H3N2 (abbreviated for A/Hong Kong/4801/2014-H3N2 like virus), A/MI/H1N1 (A/Michigan/45/2015-H1N1), and B-split (B/Brisbane/60/2008). We assessed HAI response based on (1) the proportion who seroconverted post-vaccination for each vaccine strain, as defined by at least a 4-fold rise in HAI titer at D28 compared to baseline with a titer of at least 1:40, and (2) D28/baseline log2 HAI fold rise. We explored the proportion of participants seroprotected (titer of ≥1:40) at D28 compared to baseline, but due to high baseline seroprotection rates for two of the three vaccine strains, the proportion seroprotected at D28 is a less meaningful outcome for our study objectives.

Clinically relevant variables were discussed among clinicians and based on a literature review before identifying possible factors of antibody fold rise. These included variables of interest in the aims of this study, such as metabolic co-morbidities (diabetes mellitus, hypertension, hyperlipidemia), chronic pulmonary disease, baseline 25-(OH) D, anthropometric measures of obesity (waist circumference, hip circumference, and body-mass index), demographic variables (age, gender, ethnicity), physical activity, baseline HAI titer, and prior influenza vaccine history.

Descriptive statistics were performed, and differences in patient characteristics between seroconversion status for each strain’s HAI responses were assessed based on Mann–Whitney U-test for continuous variables and Chi-square test (or Fisher’s exact test when appropriate) for categorical variables. Due to the nature of a two-fold serial dilution performed to measure antibody titer, the resulting antibody fold rise can be expressed in the form of an exponential expression. As such, the exponent in the expression for each HAI fold rise was used as outcome measurements by calculating log_2_ [HAI fold rise] to restrict the range and ensure the normality of data^47^. Multiple linear regression was then performed on each of these outcome measurements to adjust for potential confounding due to all other included variables. Similarly, the exponent in the exponential expression for each baseline HAI titer was also used as the independent variable. Test of the trend was conducted for the ordinal variables where appropriate, by treating it as a categorical variable represented as ordered integers in the regression model. Modifying effects of prior vaccination with different influenza vaccines on HAI responses were also investigated with regression models, which included interaction terms between the vaccine type and baseline HAI titer. All analyses were performed in R3.6.2, and statistical significance was deemed when a *p* < 0.05 (two-sided) was achieved^48^.

### Reporting summary

Further information on research design is available in the Nature Research Reporting Summary linked to this article.

## Supplementary information

Supplementary Information

REPORTING SUMMARY

## Data Availability

The de-identified data that support the findings of this study are available from the corresponding author upon request.
