# Supplementary material for: DYNAMIC cohort study evaluating metabolic predictors of influenza vaccine immune response in older adults
Source: NPJ Vaccines. 2022 Nov 1;7:135. doi: 10.1038/s41541-022-00548-z (PMC9626497; doi:10.1038/s41541-022-00548-z)
Supplement: Supplementary file 1 — Supplementary Information [file 41541_2022_548_MOESM1_ESM.pdf]

Supplementary Information

Contents

A. Community study sites
B. Supplementary Table 1: Multivariable regression analyses for A/HK/H3N2, A/MI/H1N1 and BSplit HAI fold rise at D28 compared to D0, inclusive of the interaction terms between prior influenza vaccination and baseline HAI titer.
C. Seasonal influenza vaccine composition in recent years
D. Supplementary Table 2: Sample size calculation table

A. Community study sites

1. Macpherson Zone “A” Residential Committee centre
2. Macpherson Zone “C” Residential Committee centre
3. Tembusu Senior Activity Centre
4. TOUCH Senior Activity Centre
5. Teck Ghee Zone “A” Residential Committee centre
6. Teck Ghee Zone “C” Residential Committee
7. Teck Ghee Zone “F” Residential Committee
8. Teck Ghee Zone “J” Residential Committee centre
9. Tampines Palmspring Residential Committee centre
10. Hua Mei Mobile Clinic
11. Telok Blangah Residential Committee
12. Ang Mo Kio Community Centre
13. Geylang West Community Centre
14. Sikh Welfare Council
15. Redhill Senior Activity Centre

B. Supplementary Table 1: Multivariable regression analyses for A/HK/H3N2, A/MI/H1N1 and BSplit HAI fold rise at D28 compared to D0, inclusive of the interaction terms between prior influenza vaccination and baseline HAI titer.

| Variables | A/HK/H3N2 Foldrise (95% CI) | p | A/MI/H1N1 Foldrise (95% CI) | p | B-Split Foldrise (95% CI) | p |
|---|---|---|---|---|---|---|
| Age | 0.99 (0.95 - 1.02) | 0.373 | 0.96 (0.92 - 0.99) | 0.025 | 1.00 (0.98 - 1.02) | 0.957 |
| Gender | | | | | | |
| Female | Ref | | Ref | | Ref | |
| Male | 0.73 (0.52 - 1.02) | 0.068 | 0.75 (0.50 - 1.13) | 0.175 | 1.03 (0.84 - 1.27) | 0.775 |
| Race | | | | | | |
| Chinese | Ref | | Ref | | Ref | |
| Malay | 1.07 (0.70 - 1.65) | 0.758 | 1.38 (0.82 - 2.31) | 0.226 | 0.96 (0.74 - 1.24) | 0.736 |
| Others | 1.27 (0.80 - 2.02) | 0.306 | 1.59 (0.91 - 2.77) | 0.104 | 1.28 (0.97 - 1.69) | 0.083 |
| Waist/ Hip/ BMI | 1.23 (0.85 - 1.78) | 0.269 | 1.33 (0.85 - 2.07) | 0.209 | 1.10 (0.88 - 1.37) | 0.418 |
| Diabetes | 1.15 (0.81 - 1.63) | 0.429 | 1.19 (0.78 - 1.80) | 0.424 | 1.00 (0.81 - 1.23) | 0.999 |
| Hypertension | 0.88 (0.62 - 1.27) | 0.503 | 0.88 (0.57 - 1.35) | 0.549 | 0.95 (0.76 - 1.18) | 0.618 |
| Hyperlipidemia | 0.90 (0.63 - 1.30) | 0.587 | 0.70 (0.45 - 1.09) | 0.114 | 1.03 (0.82 - 1.28) | 0.820 |
| Chronic Pulmonary Disease | 0.53 (0.26 - 1.11) | 0.095 | 0.78 (0.32 - 1.89) | 0.585 | 0.91 (0.59 - 1.42) | 0.694 |
| Physical Activity | | | | | | |
| Rare | Ref | - | Ref | - | Ref | - |
| Light | 2.19 (1.15 - 4.18) | 0.019 | 1.01 (0.47 - 2.20) | 0.978 | 0.99 (0.67 - 1.46) | 0.949 |
| Intermediate | 2.74 (1.25 - 6.02) | 0.013 | 0.66 (0.26 - 1.71) | 0.397 | 1.13 (0.70 - 1.81) | 0.621 |
| Moderate | 3.11 (1.57 - 6.18) | 0.001 | 0.67 (0.29 - 1.52) | 0.340 | 0.95 (0.63 - 1.44) | 0.807 |
| Vigorous | 3.29 (1.38 - 7.85) | 0.008 | 0.88 (0.31 - 2.50) | 0.808 | 0.59 (0.35 – 1.00) | 0.053 |
| Baseline 25-(OH)-D | 1.01 (0.98 - 1.03) | 0.680 | 1.00 (0.96 - 1.03) | 0.805 | 1.00 (0.99 - 1.02) | 0.610 |
| History of Influenza vaccination | | | | | | |
| None | Ref | | Ref | | Ref | |
| SH14_NH1415/ SH1516_NH1516 | 0.04 (0 - 1.43) | 0.079 | 0.02 (0 - 1.48) | 0.076 | 0.28 (0.03 - 2.48) | 0.252 |
| SH16_NH1617 | 0.21 (0.04 - 1.11) | 0.068 | 0.71 (0.09 - 5.33) | 0.738 | 0.88 (0.32 - 2.43) | 0.806 |
| Baseline AHK titer (Log2 Transformed) | 0.61 (0.54 - 0.69) | <0.001 | 1.02 (0.89 - 1.18) | 0.745 | 1.08 (1.00 - 1.16) | 0.043 |
| Baseline AMI titer (Log2 Transformed) | 0.84 (0.67 - 1.06) | 0.140 | 0.87 (0.66 - 1.15) | 0.332 | 0.93 (0.81 - 1.07) | 0.323 |
| Baseline BSplit titer (Log2 Transformed) | 1.05 (0.78 - 1.41) | 0.738 | 0.92 (0.65 - 1.31) | 0.661 | 0.81 (0.67 - 0.96) | 0.018 |
| SH14_NH1415/ SH1516_NH1516: Baseline AHK titer (Log2 Transformed) | 1.31 (0.91 - 1.89) | 0.142 | 1.31 (0.85 - 2.02) | 0.228 | 0.95 (0.76 - 1.18) | 0.651 |
| SH16_NH1617: Baseline AHK titer (Log2 Transformed) | 1.24 (1.01 - 1.52) | 0.042 | 0.98 (0.77 - 1.25) | 0.863 | 0.94 (0.83 - 1.06) | 0.323 |
| SH14_NH1415/ SH1516_NH1516: Baseline AMI titer (Log2 Transformed) | 1.61 (0.87 - 2.97) | 0.127 | 0.91 (0.44 - 1.89) | 0.795 | 1.01 (0.70 - 1.45) | 0.973 |
| SH16_NH1617: Baseline AMI titer (Log2 Transformed) | 1.08 (0.80 - 1.47) | 0.608 | 0.75 (0.52 - 1.09) | 0.131 | 1.17 (0.97 - 1.40) | 0.106 |
| SH14_NH1415/ SH1516_NH1516: Baseline BSplit titer (Log2 Transformed) | 1.84 (0.58 - 5.89) | 0.305 | 3.25 (0.81 - 13.1) | 0.099 | 1.48 (0.73 - 2.98) | 0.277 |
| SH16_NH1617: Baseline BSplit titer (Log2 Transformed) | 1.02 (0.65 - 1.61) | 0.929 | 1.30 (0.75 - 2.24) | 0.355 | 0.99 (0.75 - 1.30) | 0.922 |

Legends for ¶, †, and "a" are the same as appear in footnote for Table 1

All p values obtained by testing if the coefficient for a particular level of variable is significantly different compared to the reference level chosen for that variable after adjustment for all other variables in the regression model.

C. Seasonal influenza vaccine composition in recent years
(source: World Health Organization).

| Season/Year | Influenza A/H1N1 | Influenza A/H3N2 | Influenza B |
|---|---|---|---|
| SH 2017 (study vaccine) | A/Mich/45/2015 | A/HK/4801/2014 | B/Brisbane/60/2008 |
| NH 2016-2017 | A/California/7/2009 | A/HK/4801/2014 | B/Brisbane/60/2008 |
| SH 2016 | A/California/7/2009 | A/HK/4801/2014 | B/Brisbane/60/2008 |
| NH 2015-2016 | A/California/7/2009 | A/Switzerland/9715 293/2013 | B/Phuket/3073/2013 |
| SH 2015 | A/California/7/2009 | A/Switzerland/9715 293/2013 | B/Phuket/3073/2013 |
| NH 2014-2015 | A/California/7/2009 | A/Texas/50/2012 | B/Massachusetts/2/2012 |
| SH 2014 | A/California/7/2009 | A/Texas/50/2012 | B/Massachusetts/2/2012 |
| NH 2013-14 | A/California/7/2009 | Cell propagated prototype virus A/Victoria/361/2011 | B/Massachusetts/2/2012 |
| SH 2013 | A/California/7/2009 | A/Victoria/361/2011 | B/Wisconsin/1/2010 |
| NH 2012-2013 | A/California/7/2009 | A/Victoria/361/2011 | B/Wisconsin/1/2010 |
| SH 2012 | A/California/7/2009 | A/Perth/16/2009 | B/Brisbane/60/2008 |
| NH 2011-12 | A/California/7/2009 | A/Perth/16/2009 | B/Brisbane/60/2008 |

D. Supplementary Table 2: Sample size calculation table.

| alpha | power | N | n_non 25-(OH) D deficient | n_ 25-(OH)D deficient | N ratio | delta | p_non- 25(OH) D deficient | p_25-(OH)D deficient |
|---|---|---|---|---|---|---|---|---|
| 0.05 | 0.80 | 237 | 182 | 55 | 0.3 | -0.20 | 0.40 | 0.20 |
| 0.05 | 0.80 | 437 | 336 | 101 | 0.3 | -0.15 | 0.40 | 0.25 |
| 0.05 | 0.80 | 1016 | 781 | 235 | 0.3 | -0.10 | 0.40 | 0.30 |
| 0.05 | 0.80 | 4164 | 3203 | 961 | 0.3 | -0.05 | 0.40 | 0.35 |
| 0.05 | 0.80 | . | . | . | 0.3 | 0.00 | 0.40 | 0.40 |
| 0.05 | 0.80 | 156 | 120 | 36 | 0.3 | -0.25 | 0.45 | 0.20 |
| 0.05 | 0.80 | 254 | 195 | 59 | 0.3 | -0.20 | 0.45 | 0.25 |
| 0.05 | 0.80 | 463 | 356 | 107 | 0.3 | -0.15 | 0.45 | 0.30 |
| 0.05 | 0.80 | 1066 | 820 | 246 | 0.3 | -0.10 | 0.45 | 0.35 |
| 0.05 | 0.80 | 4332 | 3332 | 1000 | 0.3 | -0.05 | 0.45 | 0.40 |
| 0.05 | 0.80 | 111 | 85 | 26 | 0.3 | -0.30 | 0.50 | 0.20 |
| 0.05 | 0.80 | 166 | 127 | 39 | 0.3 | -0.25 | 0.50 | 0.25 |
| 0.05 | 0.80 | 266 | 204 | 62 | 0.3 | -0.20 | 0.50 | 0.30 |
| 0.05 | 0.80 | 480 | 369 | 111 | 0.3 | -0.15 | 0.50 | 0.35 |
| 0.05 | 0.80 | 1095 | 842 | 253 | 0.3 | -0.10 | 0.50 | 0.40 |
| 0.05 | 0.80 | 84 | 64 | 20 | 0.3 | -0.35 | 0.55 | 0.20 |
| 0.05 | 0.80 | 116 | 89 | 27 | 0.3 | -0.30 | 0.55 | 0.25 |
| 0.05 | 0.80 | 171 | 131 | 40 | 0.3 | -0.25 | 0.55 | 0.30 |
| 0.05 | 0.80 | 271 | 208 | 63 | 0.3 | -0.20 | 0.55 | 0.35 |
| 0.05 | 0.80 | 487 | 374 | 113 | 0.3 | -0.15 | 0.55 | 0.40 |
| 0.05 | 0.80 | 64 | 49 | 15 | 0.3 | -0.40 | 0.60 | 0.20 |
| 0.05 | 0.80 | 85 | 65 | 20 | 0.3 | -0.35 | 0.60 | 0.25 |
| 0.05 | 0.80 | 119 | 91 | 28 | 0.3 | -0.30 | 0.60 | 0.30 |
| 0.05 | 0.80 | 173 | 133 | 40 | 0.3 | -0.25 | 0.60 | 0.35 |
| 0.05 | 0.80 | 272 | 209 | 63 | 0.3 | -0.20 | 0.60 | 0.40 |
